# Genome-Wide Association Study of Root System Development at Seedling Stage in Rice

**DOI:** 10.3390/genes11121395

**Published:** 2020-11-25

**Authors:** Hongjia Zhang, Mar Lar San, Seong-Gyu Jang, Ja-Hong Lee, Na-Eun Kim, Ah-Rim Lee, So-Yeon Park, Fang-Yuan Cao, Joong-Hyoun Chin, Soon-Wook Kwon

**Affiliations:** 1 Department of Plant Bioscience, College of Natural Resources and Life Science, Pusan National University, Miryang 50463, Korea; hjzhangedu@outlook.com (H.Z.); sanmarlar2010@gmail.com (M.L.S.); sgjang0136@gmail.com (S.-G.J.); jhlp0921@gmail.com (J.-H.L.); skdms18@naver.com (N.-E.K.); aar5430@gmail.com (A.-R.L.); 2 National Institute of Crop Science, Rural Development Administration, Miryang 50463, Korea; f55261788@gmail.com; 3 Key Laboratory of Silkworm and Mulberry Genetic Improvement, Ministry of Agriculture, School of Biology and Technology, Jiangsu University of Science and Technology, Zhenjiang 212008, China; NO.1lvtu@outlook.com; 4 Department of Integrative Biological Sciences and Industry, Sejong University, 209 Neungdong-ro, Gwangjin-gu, Seoul 05006, Korea

**Keywords:** genome-wide association study (GWAS), SNP, root development, haplotype

## Abstract

Root network structure plays a crucial role in growth and development processes in rice. Longer, more branched root structures help plants to assimilate water and nutrition from soil, support robust plant growth, and improve resilience to stresses such as disease. Understanding the molecular basis of root development through screening of root-related traits in rice germplasms is critical to future rice breeding programs. This study used a small germplasm collection of 137 rice varieties chosen from the Korean rice core set (KRICE_CORE) to identify loci linked to root development. Two million high-quality single nucleotide polymorphisms (SNPs) were used as the genotype, with maximum root length (MRL) and total root weight (TRW) in seedlings used as the phenotype. Genome-wide association study (GWAS) combined with Principal Components Analysis (PCA) and Kinship matrix analysis identified four quantitative trait loci (QTLs) on chromosomes 3, 6, and 8. Two QTLs were linked to MRL and two were related to TRW. Analysis of Linkage Disequilibrium (LD) decay identified a 230 kb exploratory range for detection of candidate root-related genes. Candidates were filtered using RNA-seq data, gene annotations, and quantitative real-time PCR (qRT-PCR), and five previously characterized genes related to root development were identified, as well as four novel candidate genes. Promoter analysis of candidate genes showed that *LOC_Os03g08880* and *LOC_Os06g13060* contained SNPs with the potential to impact gene expression in root-related promoter motifs. Haplotype analysis of candidate genes revealed diverse haplotypes that were significantly associated with phenotypic variation. Taken together, these results indicate that *LOC_Os03g08880* and *LOC_Os06g13060* are strong candidate genes for root development functions. The significant haplotypes identified in this study will be beneficial in future breeding programs for root improvement.

## 1. Introduction

Rice (*Oryza sativa* L.) is one of the most significant staple food crops in the world, supporting the major energy requirements of more than half the global population [1]. Rapid societal developments have led to rice becoming a model economic crop plant, and rice yield and quality are closely associated with economic and political stability in many developing countries [2]. However, societal development is accompanied by increases in building and concomitant decreases in agricultural land availability. Moreover, climate deterioration, increasing of global temperature, and abiotic stresses, such as cold, drought, and salinity, result in the decreases in rice yield and quality [3,4,5]. Rice breeding programs to improve yield and quality are crucial to meet these challenges [6].

Rice has diverse tissues that perform different functions [7]. Root tissues play vital roles in plant growth and transport of water and mineral nutrition [8]. When plants suffer drought or other stresses, the adaptable root system supports elongation growth to obtain more water from deeper soil [9], mediated by auxin or other phytohormone responses [10]. Rice, as a monocotyledonous model plant, has root structures distinct from those of dicotyledonous models such as *Arabidopsis thaliana* [11]. In Arabidopsis, relatively few lateral roots are generated from the primary root. In rice, major functions are performed by the crown root instead of the primary root. The crown root generates the main lateral root, which then divides into larger and smaller lateral roots [12]. These advanced lateral roots are critical for the acquisition of moisture and nutrients and are highly adaptable to changes in the environment.

Rice growth encompasses four stages: germination, vegetative growth, reproductive growth, and maturity [13]. Root development has substantially different functions during these four stages. During the germination stage, the primary root mainly absorbs moisture and provides structural support [14]. During the vegetative stage, primary roots elongate to increase moisture absorption and root structure becomes more mature, with the crown root rather than the primary root acquiring nutrients from soil and supporting tillering [15]. During the reproductive and mature stages, seed setting rate, spikelet number, and seed weight, and therefore yield, are influenced by lateral root function [16]. However, root-shoot ratio has the opposite effects: increases in root content and root metabolism consume more photosynthetic resources, with negative effects on grain-filling and yield [16].

Research into root development has advanced considerably in the past two decades [17,18,19]. The plant hormones auxin and cytokinin were shown to have important roles in the development of root systems [20], and the first auxin root regulatory factor to be identified was *Crown rootless1/Adventitious rootless1* (*CRL1/ ARL1*). *CRL1/ARL1* was characterized as a novel transcription factor, containing an AS2/LOB domain, that responded to auxin and mediated the auxin signaling pathway to positively regulate crown root development in rice. *CRL1/ARL1* mutants showed decreased numbers of lateral roots, reduced sensitivity for auxin, and reduced gravitropism [21,22]. Other regulatory factors such as OsPIN family factors *OsPIN1a* and *OsPIN1b*, which were shown to modulate auxin transport [23,24], exhibited conserved sequences. A *pin1a/pin1b* CRISPR/Cas9 double mutant had shorter primary roots, smaller crown roots, and reduced gravitropism. However, the phenotypes of *pin1a* or *pin1b* CRISPR/Cas9 single mutants did not differ from the wild type [25]. The CRL4/OsGNOM1 protein was shown to assist with polarity positioning of OsPIN1 [26]. *OsCAND1* was found to be homologous to Arabidopsis *CADN1*, which participated in the development of crown root primordia via the auxin signaling pathway [27]. Overexpression of microRNA393a (miR393a) or miR393b resulted in decreased expression of auxin receptor genes *OsTIR1* and *OsAFB2*, which disturbed the auxin signaling pathway and hampered primary crown root development [28,29]. In addition to auxin-related factors, cytokinin response factors were also found to regulate or influence root system development via cytokinin response pathways [30,31,32,33]. Expression of the metallothionein gene *OsMT2b* was induced by cytokinin, and overexpression led to increased adventitious root growth, larger lateral root development, and decreased cytokinin content. An RNA interference (RNAi) line showed opposite effects, indicating that OsMT2b was involved in cytokinin regulation [34]. The key regulator of adventitious root development, OsWOX11, was shown to interact with ERF3 protein, leading to expression repression of the cytokinin response factor OsRR2 and promotion of crown root growth [35].

Genome-wide association studies (GWAS) can be used for the efficient discovery of genomic loci associated with phenotypic traits [36]. The rapid advancement of next-generation sequencing (NGS) technologies has allowed single nucleotide polymorphisms (SNPs), rather than simple sequence repeat markers, to become the marker of choice for use in genotyping studies with GWAS [37]. The large amounts of data that can be accumulated using NGS allows comprehensive genome coverage and confident SNP identification. Several GWAS studies have been published that accurately detected variation in cultivars and phenotypes [38,39].

In this study, 137 rice cultivars were collected and their maximum root length and total root weight at the two-leaf stage were assessed. GWAS analysis of root traits involving two million high-quality SNPs, Principal Component Analysis (PCA), and kinship matrices, identified five previously characterized and four novel genes as candidates for root-related traits. Promoter sequence analysis indicated that two of the candidate genes contained noteworthy SNPs in root-related motifs, and haplotype analysis of these genes showed natural variation associated with rice subspecies.

## 2. Materials and Methods

### 2.1. Plant Materials and Whole Genotype Collection

In total, 137 rice accessions were collected and used in this study (Supplementary Table S1): 19 tropical japonica, 62 temperate japonica, 43 indica, eight aus, three aromatic, and two admixture. Rice accessions were collected from 28 countries and were selected from 25,604 rice accessions in the Genebank Information Center of the Rural Development Administration (RDA-Genebank, Republic of Korea) [40].

The rice accessions were sequenced using an Illumina HiSeq 2500 Sequencing Systems Platform (Illumina Inc., San Diego, CA, USA). Average genome coverage was 8× and filtered reads were aligned to the rice reference genome (IRGSP 1.0). The following parameters were used to filter genotypes for GWAS: minor allele frequency (MAF) > 5%, missing data < 1%, and heterozygosis ratio < 5%, as implemented using Plink software [41]. Finally, approximately 2 million SNPs were selected from 6.5 million raw data SNPs.

### 2.2. Evaluation of Root System Development

To evaluate the root system, the 137 rice accessions were planted in 96-well PCR plates and cultivated in a hydroponics system, with three replicates in random arrangements. Ten seeds were used per accession for each replicate. Seed dormancy was broken by incubation at 50 °C for 4 days, after which seeds were sterilized for 10 min with 10% hypochlorite and then washed with double distilled water three times. Seeds were germinated at 28 °C for 3 days on filter paper in Petri dishes and then moved to 96-well PCR plates for growth to the seedling stage. PCR plates were incubated at 28 °C/26 °C, 12 h/12 h (day/night) at 70% humidity. Yoshida culture solution was used and was replaced daily during seedling growth [42].

Seedlings were assessed after 10 days of incubation. Maximum root length (MRL) was measured in the ten seedlings for each accession. Outlier measurements were discarded, and the average length of the remaining seedlings was determined. The average values from each of the three replicate experiments were combined to produce a final phenotype value. Next, whole seedling roots were dried at 65 °C for 5 days and used to determine total root weight (TRW). As with root length, average root weights were used to determine a phenotype value [43]. Correlation analysis was performed using Origin software to detect correlation of MRL and TRW. Bar plots showed differences according to rating levels, and box plots showed variance between diverse subgroups.

### 2.3. Population and Genotype Analysis

A series of genotype analyses were performed to complement the GWAS analysis and detect differences between populations. Population structure analysis was conducted using ADMIXTURE [44], with subgroups assigned according to delta K value, and results were visualized using Pophelper [45]. Plink software was used to generate a PCA matrix, and the optimized number (6) of principal components (PCs) was used as a Q-matrix for GWAS correction. PCA plot visualization was performed in R. Neighbor-joining trees (NJ-Tree) were generated using MEGA X [46], with the Newick format file output used for modified visualization in the online tool Interactive Tree of Life (iTOL) [47]. Linkage disequilibrium (LD) decay analysis to identify candidate regions was performed using PopLDdecay [48]. The average *R*^2^ values of pairwise SNP markers were calculated for all SNPs in the genome, and the candidate region was identified where average *R*^2^ decreased to half of the maximum value.

### 2.4. GWAS Analysis

GWAS analysis and plot visualization was performed using the GAPIT package (version 3.0) in R [49]. The Fixed and random model Circulating Probability Unification (FarmCPU) model was adopted, with PCA (Q-matrix) and kinship (K-matrix) as covariates, to correct GWAS. Due to the fact that many SNPs contain strong LD in genotype data, the thresholds decided by the total number of SNPs were too rigorous for detection of association loci [50], thus the genotype was filtered by software PLINK. Non-independence SNPs were removed and a total of 97,469 effective and independent SNPs remained, association threshold was calculated by the formula: “−log_10_ (*1/number of independent SNPs*)” [51]. Finally, the threshold was set as −log_10_(*P*) = 4.989 for identification of association loci, and SNP markers located at locus peaks were designated as lead SNPs for the detected loci. LD decay analysis identified 230 kb around the lead SNPs as candidate genomic regions for gene identification.

### 2.5. Bioinformatics Analysis and Candidate Gene Prediction

Candidate genomic regions were identified from LD decay and GWAS analyses. Functional gene annotations were assigned to candidate regions from the rice genome website Gramene (www.gramene.org/). RNA-seq data from the GEO database in NCBI (www.ncbi.nlm.nih.gov) were used to assess tissue-specific expression of candidate genes. GEO accessions followed GSE6893 and GSE56463, each sample was analyzed by three replicates, respectively. Data results of root, leaf, shoot apical meristem (SAM), inflorescence, seed, mature seed from dataset GSE6893, analysis process combination with results of seeds, flower buds, flowers, flag leaves, and root before and after flowering were from dataset GSE56463. Gene ontology (GO) annotations for GO enrichment analysis were obtained from agriGO (systemsbiology.cau.edu.cn/agriGOv2). Heatmaps and bubble plots were constructed using TBtools and R, respectively [52].

### 2.6. RNA Extraction, cDNA Synthesis, and Expression Analysis

Gene expression was assessed in three rice varieties exhibiting extreme ‘favorable’ root-related traits and three exhibiting extreme ‘unfavorable’ traits. Total RNA was extracted from seedling roots using a RNeasy Plant Mini Kit (QIAGEN) and samples were treated with RNase-Free DNase (QIAGEN) to remove genomic DNA. Complementary DNA (cDNA) was synthesized using a SuperScript III Kit (Thermo), with primers for target genes designed using Primer5 (Supplementary Table S2). SYBR Green PCR Kit (QIAGEN) reagents were used for qRT-PCR analysis. Reactions were performed in triplicate, and *actin* expression was used as an internal baseline control. The −ΔΔCt method was used to calculate relative gene expression [53].

### 2.7. Sequence and Haplotype Analysis

Sequence and haplotype analysis were performed for each gene to assess genetic and subspecies variation. For haplotype analysis, all SNP markers from the gene were used, but missing and heterozygote data were excluded. Average score and variety count were determined from phenotype data for each subspecies, and haplotypes were identified that were significantly associated with phenotype. LD analysis was conducted using HaploView 4.2 [54]. SNP markers that were favorable for haplotype analysis were also used in LD analysis for analysis of conservation level. Haplotype variation analysis was performed using PopART software [55]. The online tool Gene Structure Display Server 2.0 [56] was used for visualization of gene structure and SNP position.

## 3. Results

### 3.1. Phenotype Evaluation of Root System Development

**Figure 1:**
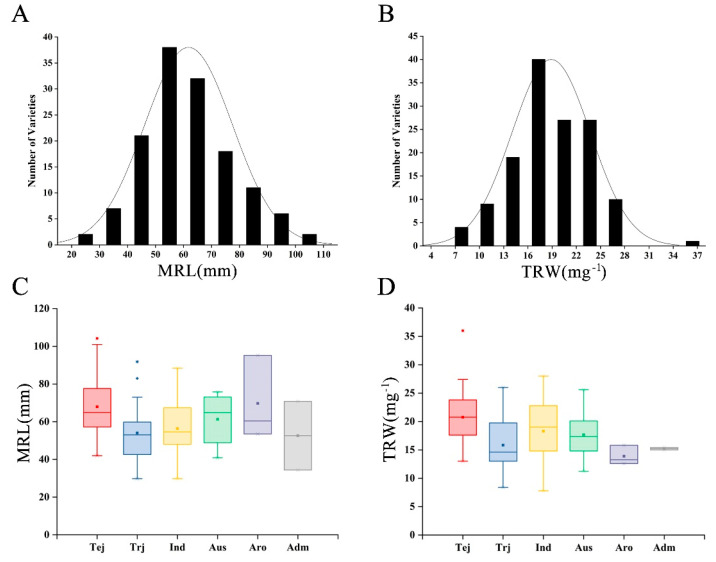
Evaluation and statistical analysis of root system development in 137 rice varieties. (**A**) Evaluation of maximum root length (MRL). (**B**) Evaluation of total root weight (TRW). (**C**) Statistical analysis of maximum root length (MRL) according to rice subspecies. (**D**) Statistical analysis of total root weight (TRW) according to subspecies. Tej: temperate japonica, Trj: tropical japonica, Ind: indica, Aro: aromatic, Adm: admixture. Box plot: solid lines within boxes represent median values.

Six rice subspecies encompassing 137 rice varieties were used for assessment of root growth and development: Tropical japonica (Trj), Temperate japonica (Tej), Indica (Ind), Aus, Aromatic (Aro), and Admixture (Adm). Maximum root length (MRL) followed a continuous distribution with a range of 29.8–104.2 mm (Figure 1A). Total root weight (TRW) was in the 7.8–36 mg^−1^ range and also followed a continuous distribution (Figure 1B). These results indicated that MRL and TRW were quantitative traits controlled by multiple genes. Subspecies variation of MRL was assessed. The Tej group had slightly longer roots than the Aus and Aro groups, and substantially longer roots than groups Trj and Ind (Figure 1C). TRW showed similar trends, with the exception that root weight in group Ind was similar to Tej (Figure 1D).

**Figure 2:**
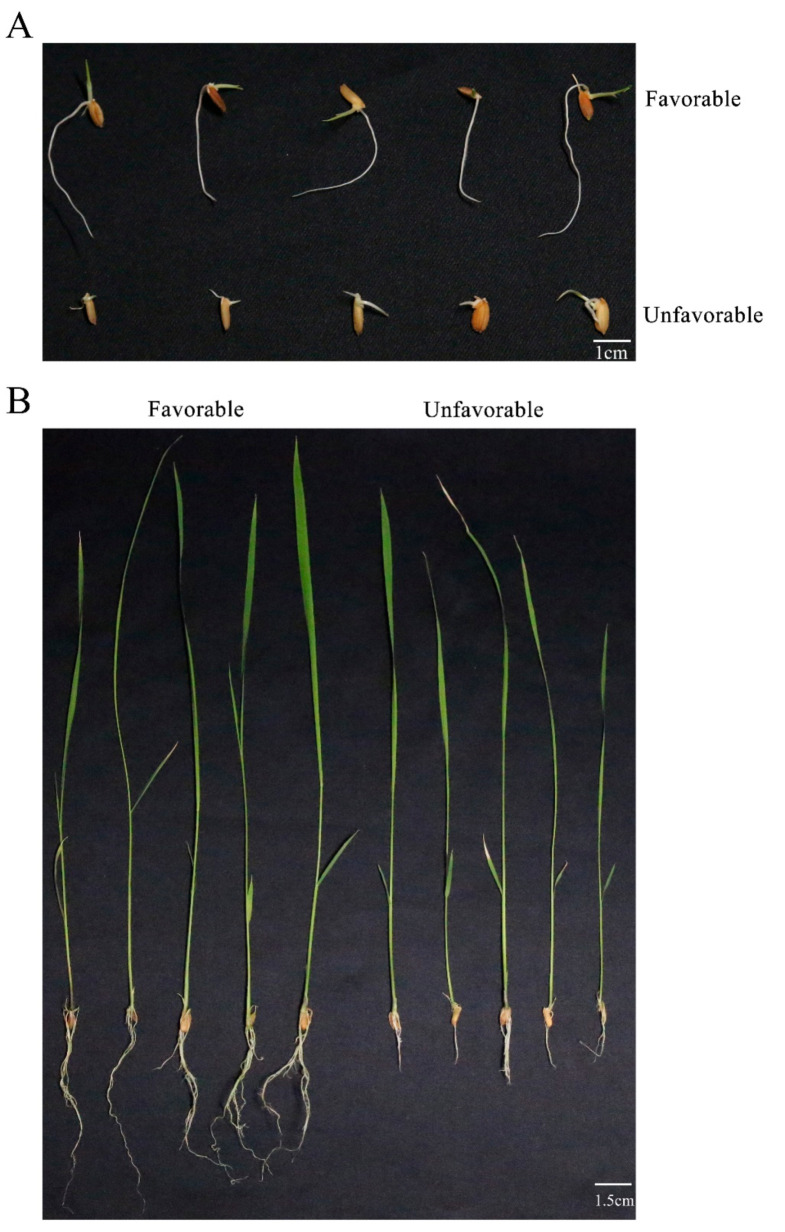
Phenotypes of rice varieties with extreme root phenotypes. (**A**) Root development at germination stage. Upper and lower rows represent ‘favorable’ and ‘unfavorable’ root development, respectively. Scale bar = 1 cm. (**B**) Root development at the seedling stage. Left and right seedlings are favorable and unfavorable varieties, respectively. Scale bar = 1.5 cm.

Five varieties with well-developed roots (‘favorable’) and five with poorly developed roots (‘unfavorable’) were examined further, and the MRL and TRW phenotypes exhibited significant differences (Figure 2B). These differences were observed during the seed germination stage as well as in the seedling stage (Figure 2A). Correlation analysis showed a slight positive correlation between MRL and TRW (Supplementary Figure S1), with a correlation coefficient of 0.206 (*p* < 0.001). These results suggested that some varieties would be able to absorb more nutrients from the earliest germination stage. The favorable root development phenotype would be predicted to support improved growth during the seedling stage and in subsequent developmental stages. Correlation between MRL and TRW suggested that some varieties benefited from root branch growth and that vigorous lateral root development was beneficial to the development of the root system.

### 3.2. Population Structure and LD Decay Analysis

**Figure 3:**
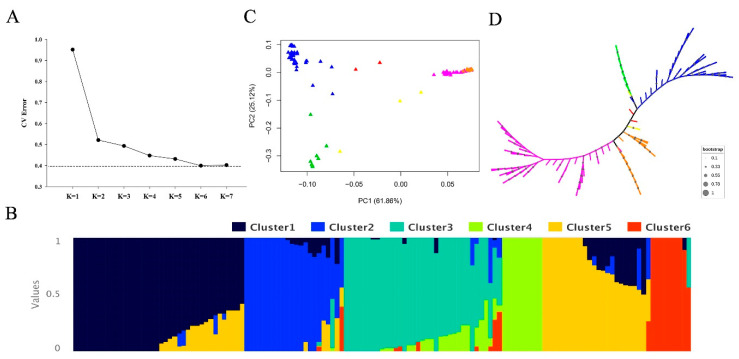
Population structure analysis. (**A**) Cross-validation (CV) error of diverse groups (K). The dotted transverse line represents the lowest level. (**B**) Structure analysis outcome (K = 6). (**C**) Principal Component Analysis (PCA) (PC1 and PC2). Pink, orange, blue, green, yellow, and red represent the Tej, Trj, Ind, Aus, Aro, and Adm rice subspecies, respectively. (**D**) NJ-Tree of the rice population. Pink, orange, blue, green, yellow, and red represent the Tej, Trj, Ind, Aus, Aro, and Adm rice subspecies, respectively. Grey dots represent bootstrap information.

Population structure was assessed using PCA and neighbor-joining tree analyses. Cross-validation (CV) analysis indicated that K = 6 was the optimal population grouping, with the lowest error ratio compared with other K values (Figure 3A). A structure plot for the K = 6 structure was generated using Pophelper (Figure 3B). As expected, most of the rice varieties were distinguished according to their subspecies (Supplementary Figure S2). PCA (total PCs = 6) of all the genotype data was performed using Plink software. PC1 and PC2 explained most of the variation (61.86% and 25.12%) and were selected for visualization. Significant clusters were observed for rice subspecies groups Tej, Trj, Ind, and Aus, whereas the Aro and Adm groups exhibited more ambiguous separation (Figure 3C). NJ-Tree analysis of genetic distance exhibited similar results, with most varieties clearly clustered by subspecies and only Adm and Aro varieties showing dispersion among diverse clusters. LD decay was analyzed in the full panel of rice varieties. The *R*^2^ declined with increasing physical distance. The threshold value for candidate regions was determined as half the maximal *R*^2^ value, 0.26, which produced a genomic candidate region of 230 kb (Supplementary Figure S3).

Taken together, the structure, PCA, and NJ-Tree analyses divided the 137 rice accessions into six optimal subgroups. With the exception of a few varieties, the majority of accessions were grouped according to their subspecies. Candidate regions for further GWAS analysis were identified.

### 3.3. GWAS Analysis

**Figure 4:**
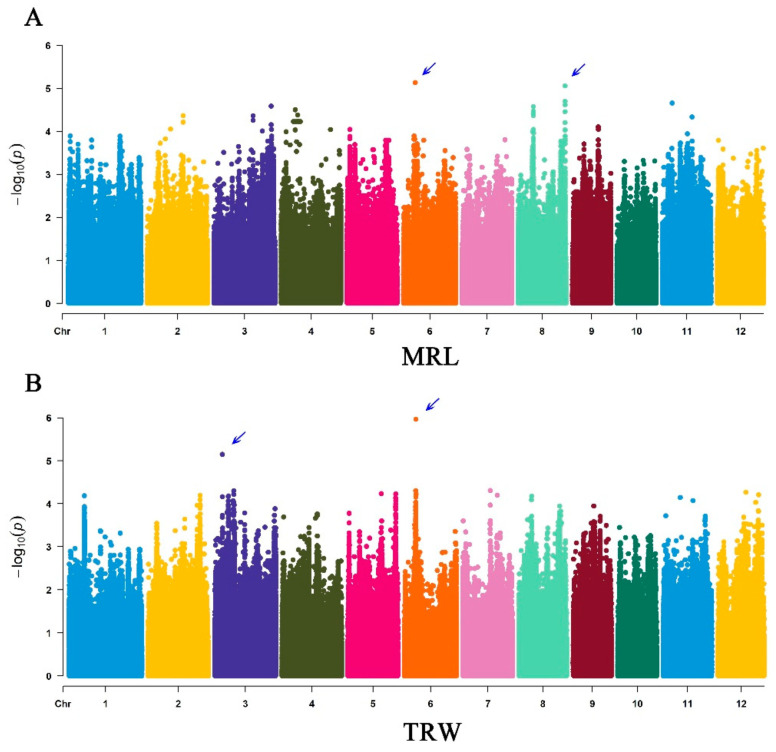
Manhattan plots for maximum root length (MRL) and total root weight (TRW) in 137 rice varieties. (**A**) GWAS of MRL using the FarmCPU model. (**B**) GWAS of TRW using the FarmCPU model. *X* axis indicates physically mapped chromosomes. *Y* axis indicates significance as calculated by −log_10_ (*P*).

GWAS analysis was performed using the GAPIT package in R. The previous results of PCA and Kinship as the Q-Matrix and K-Matrix respectively, as the covariate co-analyzed by combination with genotype and phenotype. Manhattan plots for MRL and TRW are shown in Figure 4. Four association loci, two each for MRL and TRW, were identified (Table 1). The two association loci for MRL, named *qMRL6* and *qMRL8*, were located on chromosomes 6 and 8, and had −log_10_(*P*) of 5.135 and 5.059, respectively. The two association loci for TRW, named *qTRW3* and *qTRW6*, were located on chromosomes 3 and 6 and had −log_10_(*P*) of 5.148 and 5.967, respectively. LD decay analysis indicated that the MRL and TRW loci on Chr6 were in close proximity, with a significant overlapping region. The distance between the lead SNPs (chr06_7017401 and chr06_6908583) was approximately 108 kb. The association loci were searched for relevant genes, and five previously characterized genes related to root development were identified. Of these, *OsMADS47, OsAFB6*, and *PIN1a* were previously characterized as positive regulators of root development [25,57,58], and *AHP1* and *OsGA20ox7* were found to negatively regulate root development [59,60]. Overall, this analysis identified four association loci associated with root development and identified several genes responsible for root development traits. Additional gene analysis suggested that several uncharacterized genes in the association loci might also be related to root development.

### 3.4. Candidate Genes and Bioinformatics Analysis

**Figure 5:**
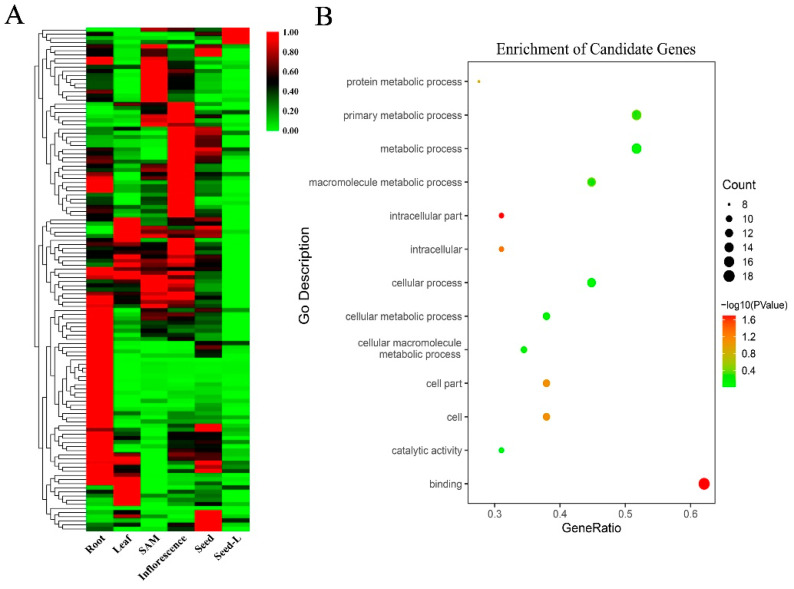
Analysis of candidate root-related genes. (**A**) Gene expression in different tissues using RNA-seq data. Red and green represent the high and low expression, respectively. Seed-L represents mature seed. (**B**) GO enrichment analysis of 44 candidate genes.

The GWAS analysis detected four association root-related loci on chromosomes 3, 6, and 8. Analysis of candidate genes from the association loci was conducted using RNA-seq data. The LD decay results focused the search for novel functional genes to a 230 kb region. For gene annotations, hypothetical proteins or non-protein coding transcripts were filtered, and then RNA-seq data were used to assess gene expression in different tissues [61,62]. Partial results are shown in Figure 5A. Genes were identified that showed significantly different expression in roots compared with other tissues and, alongside examination of gene annotations or other references studied, this allowed the identification of 44 candidate genes (Supplementary Table S3). GO enrichment analysis showed that most of the candidate genes were involved in binding and metabolic process functions (Figure 5B). Examination of published references, gene family descriptions, and homologous genes on other species was used to further refine the list of candidate genes. Finally, seven candidate genes were selected from three association loci (two loci and the overlap region).

**Figure 6:**
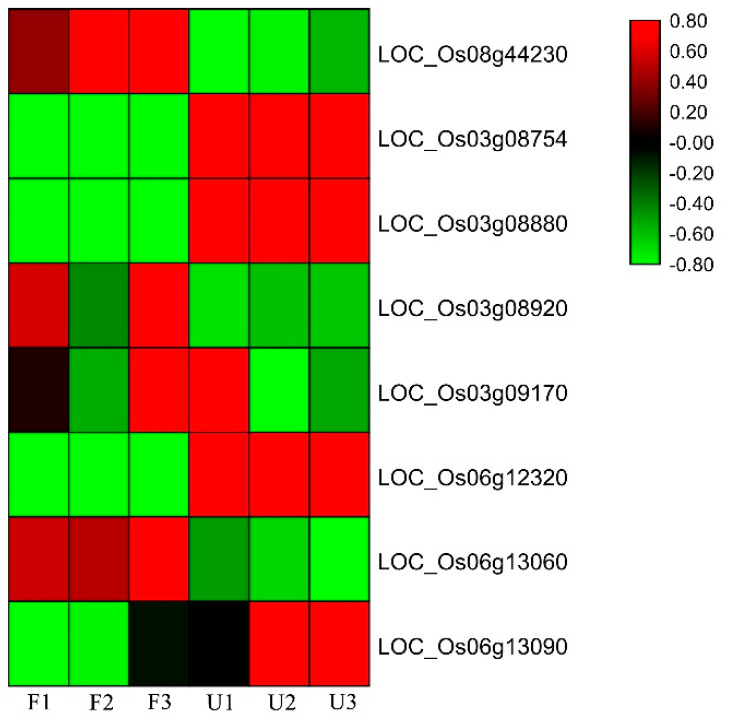
Analysis of candidate genes in varieties with ‘favorable’ and ‘unfavorable’ root development with qRT-PCR. F1, F2, and F3 represent favorable varieties, and U1, U2, and U3 represent unfavorable varieties. Three biological and three technical experimental replicates were performed. Red and green represent high and low expression, respectively.

Expression of candidate genes in rice varieties with favorable and unfavorable root traits was assessed using qRT-PCR, with the *OsMADS47* gene used as a reference. Primers are shown in Supplementary Table S2. Three favorable (F1, F2, F3) and three unfavorable varieties (U1, U2, U3) that exhibited extremes of MRL and TRW were used (Figure 6). Five genes (*LOC_Os08g44230*, *LOC_Os03g08754*, *LOC_Os03g08880*, *LOC_06g12320*, and *LOC_Os06g13060*) were differentially expressed between the favorable and unfavorable varieties. Two candidate genes (*LOC_Os08g44230* and *LOC_Os06g13060*) had higher expression levels in favorable varieties than in unfavorable varieties (Figure 6), and two candidate genes (*LOC_Os03g08880* and *LOC_06g12320*) and *OsMADS47* (*LOC_Os03g08754*) exhibited higher expression in unfavorable varieties than in favorable varieties (Figure 6). *LOC_Os08g44230* encodes a zinc finger family protein of unknown function. *LOC_Os03g08880* (*OsPUP1*) encodes a purine permease belonging to the OsPUP family. Previous research showed that *OsPUP1* was expressed at high levels in roots and that another OsPUP family member, *OsPUP7*, was involved in growth and development in rice [63]. *LOC_06g12320* encodes a transmembrane amino acid transporter of unknown function. *LOC_Os06g13060* (*OsDjC54*) encodes a heat shock protein, and its Arabidopsis homolog, *AT1G24120* (*ARL1*)*,* was shown to be involved in root development [64].

The combination of RNA-seq, GO annotation, and qRT-PCR analysis, alongside previous research, facilitated the selection of four candidate root development genes from three loci.

### 3.5. Analysis of SNP Function in Promoter Region

Promoter involvement in expression regulation prompted the examination of SNPs in the promoter regions of candidate genes. Promoter regions were considered to be the 1500 bp immediately upstream of ATG start codon, except where intergenic regions necessitated shorter promoter sequences, as in *LOC_Os03g08754, LOC_Os03g08880,* and *LOC_Os06g13060*. Five root-related promoter motifs were identified using the online tool New PLACE: auxin response factor motifs ARFAT and ASF1MOTIFCAMV, and root development or expression motifs RSEPVGRP1, RAV1AAT, and RAV1BAT.

**Figure 7:**
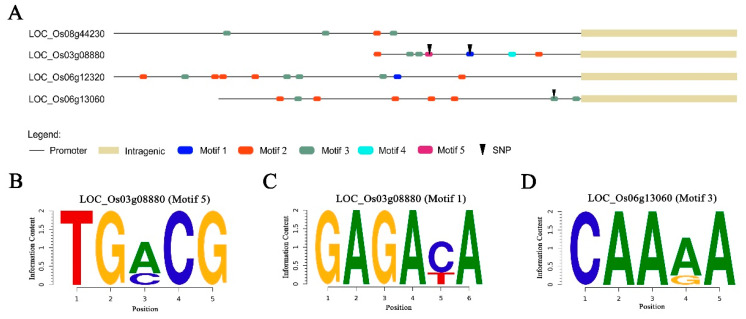
Promoter analysis of four candidate genes. (**A**) Structure of candidate genes. Structure description followed the legend, the shorter promoter depends on distance of intergenic. Motif 1: ARFAT, Motif 2: RSEPVGRP1, Motif 3: RAV1AAT, Motif 4: RAV1BAT, Motif 5: ASF1MOTIFCAMV. (**B**–**D**) Sequence logo of root-related motifs. The height of each base corresponds to its frequency of occurrence in the population.

The root-related motifs were assessed in the promoter regions of the four candidate genes (Figure 7A), and three Sequence logo showed the structure of motifs (Figure 7B,D). Two genes (*LOC_Os03g08880* and *LOC_Os06g13060*) harbored SNPs in root-related motifs. In *LOC_Os03g08880*, one SNP, A to C, was identified at promoter position 173 bp in motif 5 (ASF1MOTIFCAMV) (Figure 7B). A second SNP, C to T, was located at promoter position 309 bp in motif 1 (ARFAT) (Figure 7C). *LOC_Os06g13060* contained one SNP, A to G, at promoter position 1065 bp in motif 3 (RAV1AAT) (Figure 7D). These results suggested that genes *LOC_Os03g08880* and *LOC_Os06g13060* were likely candidates for transcriptional regulation during root development.

### 3.6. Haplotype Analysis of Reported and Novel Candidate Genes

**Figure 8:**
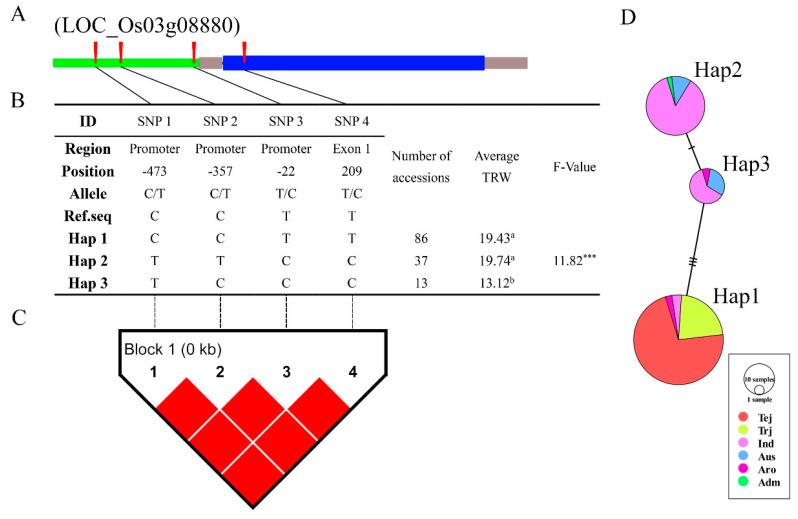
Haplotype analysis of *LOC_Os03g08880*. (**A**) Schematic representation of gene structure and SNPs positions in *LOC_Os03g08880*. Green, gray, and blue blocks represent promoter, untranslated region (UTR), and exon region, red vertical bars represent SNPs. (**B**) Results of haplotype analysis. Hap: Haplotype. Letters: a, b represent different significance level at *** *p* < 0.001 (Duncan’s test). (**C**) Linkage disequilibrium (LD) analysis of SNPs in *LOC_Os03g08880*. *D’* was used to indicate LD level, with LD blocks defined using the Confidence Intervals function in the analysis software. Red blocks indicate complete LD between each SNP. (**D**) Haplotype variation analysis. Colors indicate rice subspecies as indicated in the legend. Circle size indicates the number of varieties in each Hap. Traverse lines represent the extent of variation between two Haps.

**Figure 9:**
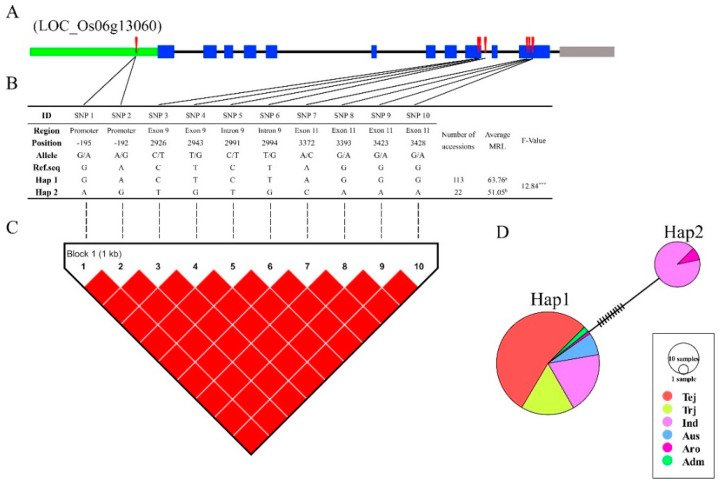
Haplotype analysis of *LOC_Os06g13060*. (**A**) Schematic representation of gene structure and SNP positions of *LOC_Os06g13060*. Green, gray, and blue blocks represent promoter, untranslated regions, and exon regions. Solid lines indicate intron regions. Red vertical bars represent SNPs. (**B**) Results of haplotype analysis. Hap: haplotype. Letters: a, b represent different significance level at *** *p* < 0.001 (Duncan’s test). (**C**) LD analysis of SNPs in *LOC_Os06g13060*. *D’* was used to indicate LD level, with LD blocks defined using the Confidence Intervals function in the analysis software. Red blocks indicate complete LD between each SNP. (**D**) Haplotype variation analysis. Colors indicate rice subspecies as indicated in the legend. Circle size indicates the number of varieties in each Hap. Traverse lines represent the extent of variation between two Haps.

In total, five characterized and two novel candidate genes were identified by GWAS. Next, haplotype analysis was performed using genotyping data. After removal of heterozygote and missing data, SNPs from promoters and intragenic regions were used for haplotype, LD, and haplotype variation analysis. Results from two candidate genes are shown in Figure 8 and Figure 9. *LOC_Os03g08880* contained four SNPs in the promoter and exon regions (Figure 8A), and the LD block showed significantly strong LD between each of the SNPs (Figure 8C). Haplotype analysis of the 136 rice varieties showed that the four SNPs divided into three haplotypes (Haps), with the maximum phenotypic TRW variation of 6.62 between Haps 2 and 3 (Figure 8B). TRW variation was significant with Hap 3 but was not significant between Haps 1 and 2. This was expected, as the major constituents of these two Haps were indica varieties (Figure 8D). *LOC_Os06g13060* contained ten SNPs in the promoter, intron, and exon regions (Figure 9A). The LD block showed a similar result with *LOC_Os03g08880*, a strong LD level between all SNPs in this gene (Figure 9C). *LOC_Os06g13060* was found in the overlapping genomic region between the MRL and TRW loci, and the ten SNPs divided into two Haps with phenotypic variations of 12.71 for MRL (Figure 9B). Hap 1, which included all the japonica and a few other varieties, showed large variation compared with Hap 2 (Figure 9D).

Four of the five characterized candidate genes exhibited significant phenotypic variation (Supplementary Figures S4–S7). With respect to LD blocks, *LOC_Os08g44350* and *LOC_Os03g08754* had high linkage levels between each SNP (Supplementary Figures S4C and S6C). *LOC_Os08g44350* and *LOC_Os08g44590* contained three and four Haps respectively, and exhibited significant phenotypic MRL variations of 28.16 and 30.04, respectively. The unfavorable Haps were comprised of indica or admixture varieties (Supplementary Figures S4B and S5B). *LOC_Os03g08754* and *LOC_Os06g12610* contained significant TRW phenotypic variations of 7.34 and 8.19, respectively (Supplementary Figures S6B and S7B). Large and complex variations were identified in six Haps for each of these genes, with clear grouping apparent for most of the japonica, indica, and other subspecies varieties. These results suggested that the root phenotype variation between diverse haplotypes emerged due to evolutionary processes.

In summary, haplotypes exhibiting significant root phenotype variation were identified for several candidate genes, including characterized and novel genes. These results will provide useful information towards rice breeding.

## 4. Discussion

### 4.1. Root System Development as the Crucial Trait for Rice

GWAS analysis was used in this study to detect association loci associated with maximum root length (MRL) and total root weight (TRW) traits in rice (Figure 1 and Figure 2). In rice, water capture, nutrient absorption, and metabolic processes are strongly influenced by root systems. The major root components, namely, the primary root, crown root, and lateral root, together comprise a complex root system, each part of which has diverse functions during different rice growth stages. For instance, primary roots are important for mineral nutrient acquisition and support shoot growth during the germination and young seedling growth stages [65]. The major functions of the crown and lateral roots are water and nutrient uptake and regulation of interactions between the plant and its environment [19].

Recent studies using GWAS or Quantitative Trait Loci (QTL) analysis identified several novel root-related QTLs. A GWAS analysis used 21 traits and 529 representative rice accessions and identified 143 significant associations, 25 of which were novel. The involvement of the multi-functional gene *Nal1* and a novel functional gene, *OsJAZ1*, in the regulation of root development was demonstrated using overexpression and RNAi transgenic rice lines [9]. A study conducted by Wang and colleagues used 307 rice accessions for GWAS analysis and identified six significant QTLs associated with maximum root length, crown root number, total root length, and root tip number traits [43]. Together, these studies suggested that maximum root length and total root weight were optimal traits for identification of candidate genes for root development. Additional factors such as root responses to abiotic stresses in natural disasters were also shown to be important for rice growth. Previous GWAS analysis involving root system and abiotic stress-related phenotypic traits allowed the identification of loci and candidate genes related to root development under salt, cold, or abscisic acid stress [9]. Use of GWAS to identify useful traits will facilitate breeding programs to develop rice varieties that are more resilient to environmental changes.

### 4.2. Candidate Genes Identified by GWAS

In this study, GWAS analysis was performed with 137 diverse rice varieties and approximately 2 million high-quality SNPs (Figure 4). PCA matrix, kinship matrix, and LD decay analysis identified four loci and 230 kb candidate regions for root-related traits (Supplementary Figure S3). RNA-seq data, gene annotations, and previous research identified 44 candidate genes in the association loci, including five previously reported genes. Further qRT-PCR analysis showed that four of the candidate genes exhibited different expression patterns in varieties with extreme ‘favorable’ and ‘unfavorable’ root-related phenotypes (Figure 6). Ordinarily, use of structure and kinship results as covariates for GWAS analysis, with structure as the population structure for classification of subspecies, can decrease GWAS false-positives [41]. The rice collection used in this study included accessions from diverse rice subspecies (Tej, Trj, Ind, etc.), and structure analysis was therefore necessary for result correction. Kinship as the genetic relationship showed similar functions to structure.

Several candidate genes were identified in the association loci. RNA-seq data was used to assess gene expression in diverse samples, as genes were more likely to have root-related functions if significant differences in expression were observed between root and other tissues (Figure 5A). Studies of gene families or homologous genes in other species were also used as evidence for determination of promising candidate genes. Eventually, seven root-related candidate genes were identified. Five of these genes, *LOC_Os08g44230*, *LOC_Os03g08880*, *LOC_Os03g08920*, *LOC_Os03g09170*, and *LOC_Os06g12320*, showed significant differences in expression between root and other tissues in two RNA-seq datasets. Arabidopsis homologs of the other two genes, *LOC_Os06g13060* and *LOC_Os06g13090*, had root-related functions in Arabidopsis. Analysis of these genes by qRT-PCR revealed that four genes, *LOC_Os08g44230*, *LOC_Os03g08880*, *LOC_Os06g12320*, and *LOC_Os06g13060*, had distinct expression patterns associated with the root traits in the diverse panel of rice accessions. These four genes were thus promising candidates for further analysis.

### 4.3. LD, Haplotype, and Functional SNP Analysis

Two of the four promising candidate genes, *LOC_Os03g08880* and *LOC_Os06g13060*, contained functional SNPs in root-related promoter motifs (Figure 7). Furthermore, haplotype analysis of these two genes and five previously characterized candidate genes revealed diverse haplotypes that were associated with phenotypic variation or related to rice subspecies. Functional cis-acting elements or motifs in the promoter regions play crucial roles in signaling pathways through the activity of transcription factors that identify and bind to specific promoter motifs of downstream target genes. Research by Inukai and colleagues showed that the *crl1* gene was the key regulator of lateral root development. Two auxin-response motifs (AuxREs) in the promoter region of *crl1* were specifically bound by auxin-response family factors, linking regulation of root development to the auxin signaling pathway [21]. Five diverse root-related motifs were identified in the promoters of the four main candidate genes. *LOC_Os03g08880* had SNPs in promoter motifs ASF1MOTIFCAMV and ARFAT, and *LOC_Os06g13060* had a SNP in the RAV1AAT motif. Changes in these motifs may have altered their binding by upstream transcription factors, which would impact gene expression and lead to positive or negative changes in the regulation of root development.

In the genome region, LD was constructed by multiple SNPs. Due to the LD degraded by the crossover between genes, therefore, LD level was dependent on genome recombination rate. In a steady population, if some SNPs that located in one region never occur recombination in the genome, these SNPs will show a strong LD level between each SNP, the gene located in this region will more likely to be prominent and conserved. Popularly, LD decay analysis is aimed to find the proper LD level for detecting candidate genes, set as when the *R*^2^ decreases to half of maximum [40]. The diversity of rice accessions meant that diverse LD levels associated with genetic variation were found across the genome. LD decay was therefore assessed across the whole genome and the physical map for the appropriate regions. Also, the LD block could show the LD level between each SNP, as well the multiple SNPs make a haplotype block by strong LD level, which is contributed to identified novel haplotype and functional genes. In Yuan’s study, they identified multiple strong LD regions in candidate regions using the diverse model and diverse subspecies [66]. Two candidate genes, *OsSTL1* and *OsSTL2,* were identified from strong LD regions in GWAS results. In the present study, as the result of LD decay analyzed, candidate genes were selected in the 230 kb region around lead SNPs, and two novel genes and four reported genes were identified. Among intragenic and promoter regions, all SNPs showed a complete strong LD level in genes *LOC_Os03g08880*, *LOC_Os06g13060*, *LOC_Os08g44350*, *LOC_Os08g44590*, and *LOC_Os03g08754*, which suggested that these genes contain a conserved function during diverse evolutionary or crossing processes. Haplotype analysis of most of the candidate genes showed clear grouping by rice subspecies, suggesting that the major groups emerged alongside the historical development of rice. Societal development, migration to new environments, and climate alterations may have increased the variation between subspecies and enhanced the variation in root phenotypes, but further research is needed to address this hypothesis.

## 5. Conclusions

In this study, maximum root length and total root weight were assessed in a panel of 137 diverse rice accessions. Four association loci associated with the root-related traits were identified by GWAS analysis. Five previously characterized genes and four novel candidate genes were identified, and SNPs in root-related promoter motifs were assessed. Haplotype analysis of the five characterized genes and two novel genes revealed diverse haplotypes associated with root phenotypic variation. This study supported beneficial information for improvement of future breeding.

## Figures and Tables

**Table 1:** Summary of genome-wide association loci for maximum root length (MRL) and total root weight (TRW).

Traits	QTLs	Lead SNPs	Chr	Position	−log_10_(*P*)	Reported Genes
MRL	*qMRL6*	chr06_7017401	6	7017401	5.135	*OsPIN1a*
	*qMRL8*	chr08_27868839	8	27868839	5.059	*OsAHP1, OsGA20ox7*
TRW	*qTRW3*	chr03_4592524	3	4592524	5.148	*OsMADS47, OsAFB6*
	*qTRW6*	chr06_6908583	6	6908583	5.967	*OsPIN1a*

## Supplementary Materials

The following are available online at https://www.mdpi.com/2073-4425/11/12/1395/s1, Figure S1: Correlation analysis of MRL and TRW, Figure S2: Information of population structure, Figure S3: LD decay analysis, Figure S4: Haplotype analysis of *LOC_Os08g44350*, Figure S5: Haplotype analysis of *LOC_Os08g44590*, Figure S6: Haplotype analysis of *LOC_Os03g08754*, Figure S7: Haplotype analysis of *LOC_Os06g12610*, Table S1: List of whole panel for this study, Table S2: List of primers for qRT-PCR, Table S3: List of tentative candidate genes identified by this study.
