# Supplementary material for: Genome-Wide Association Study of Root System Development at Seedling Stage in Rice
Source: Genes (Basel). 2020 Nov 25;11(12):1395. doi: 10.3390/genes11121395 (PMC7760126; doi:10.3390/genes11121395)
Supplement: Supplementary file 1 [file genes-11-01395-s001.zip › genes-974928-suppls/Figure S1-S7.docx]

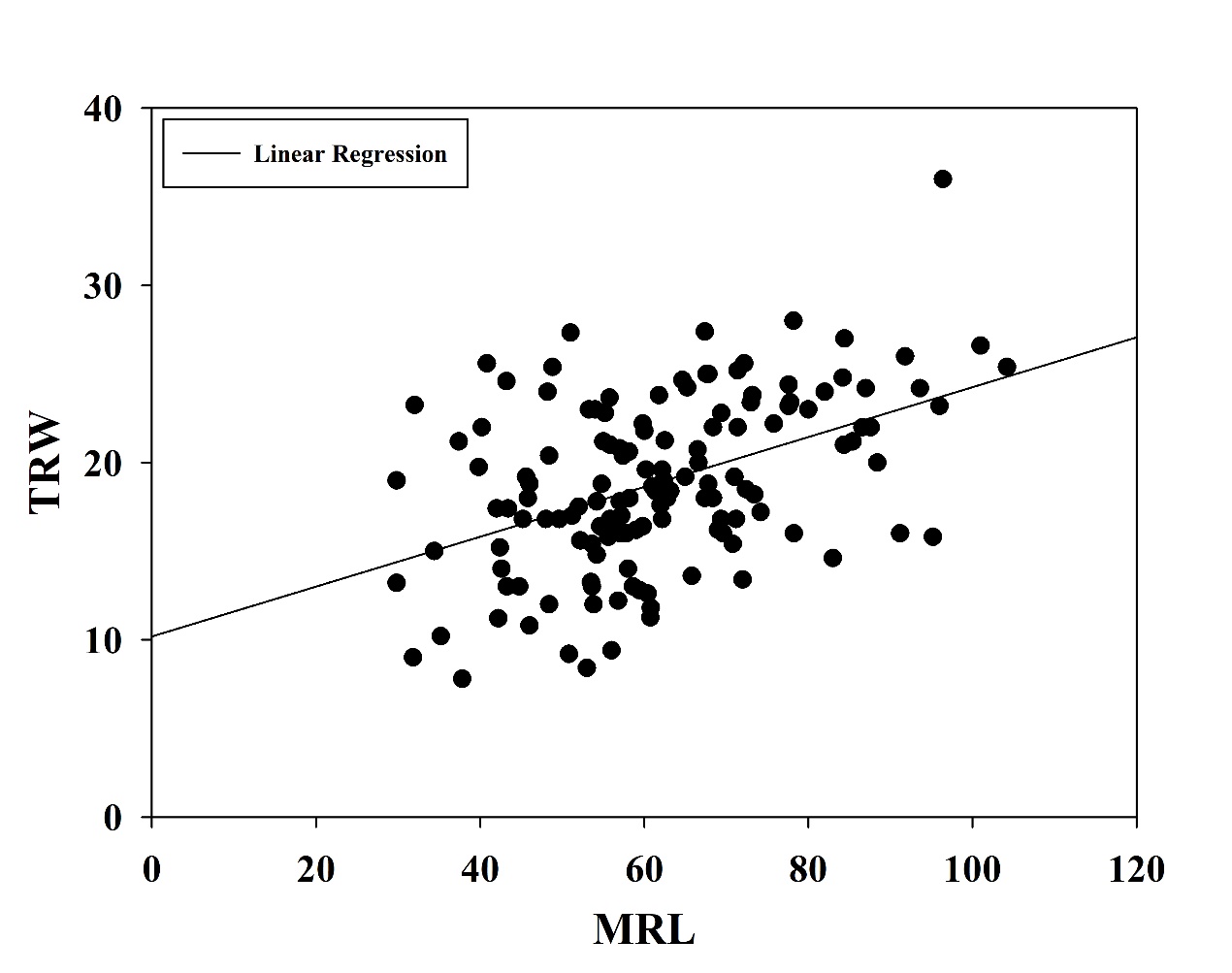


**Figure 1.** Correlation analysis of MRL and TRW. *R*^2^ = 0.206 (P<0.001).


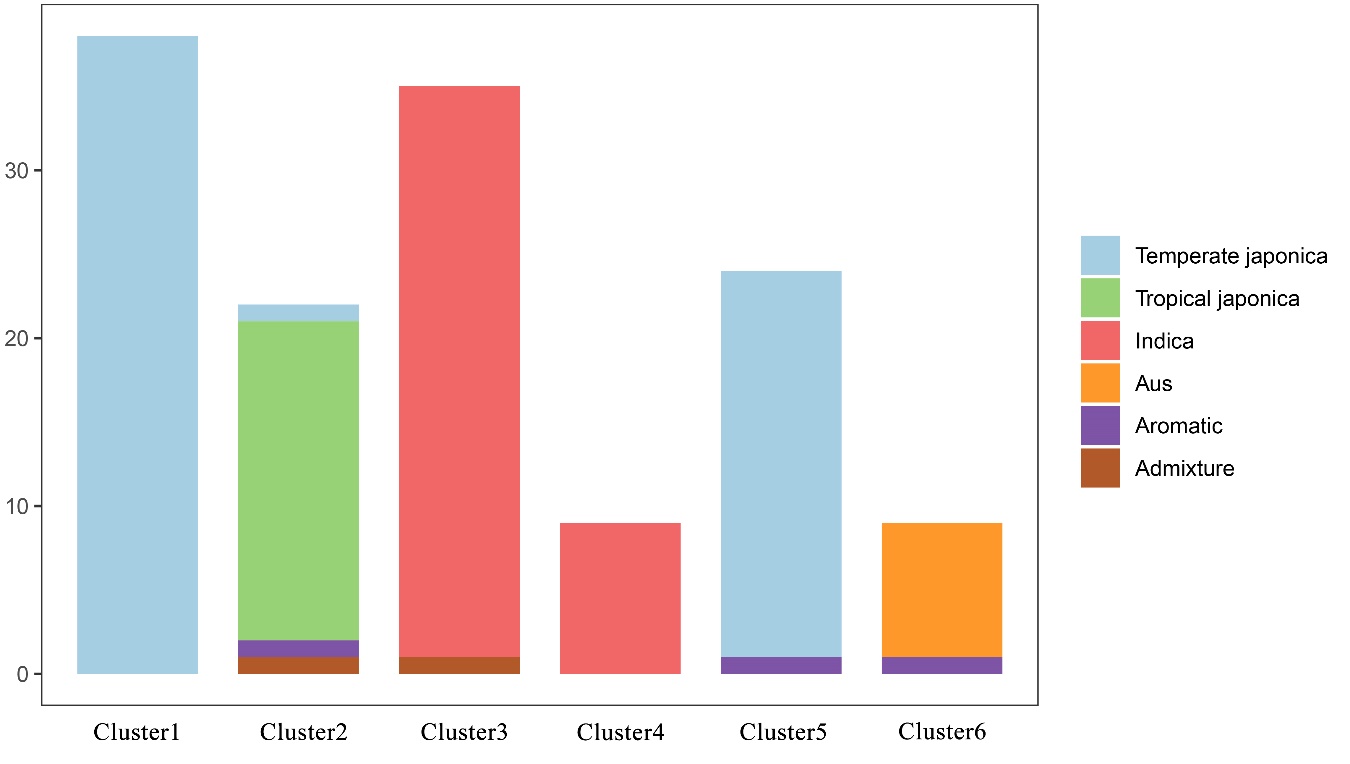


**Figure 2.** Information of population structure. X-axial and Y-axial represent six group and count of varieties in each subgroup, respectively, color information follows the legend in figure.


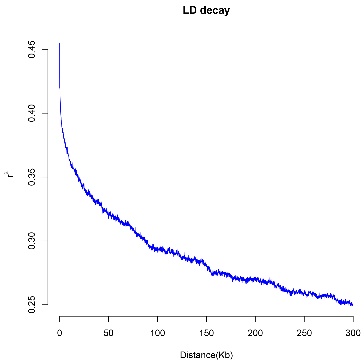


**Figure 3.** LD decay analysis.


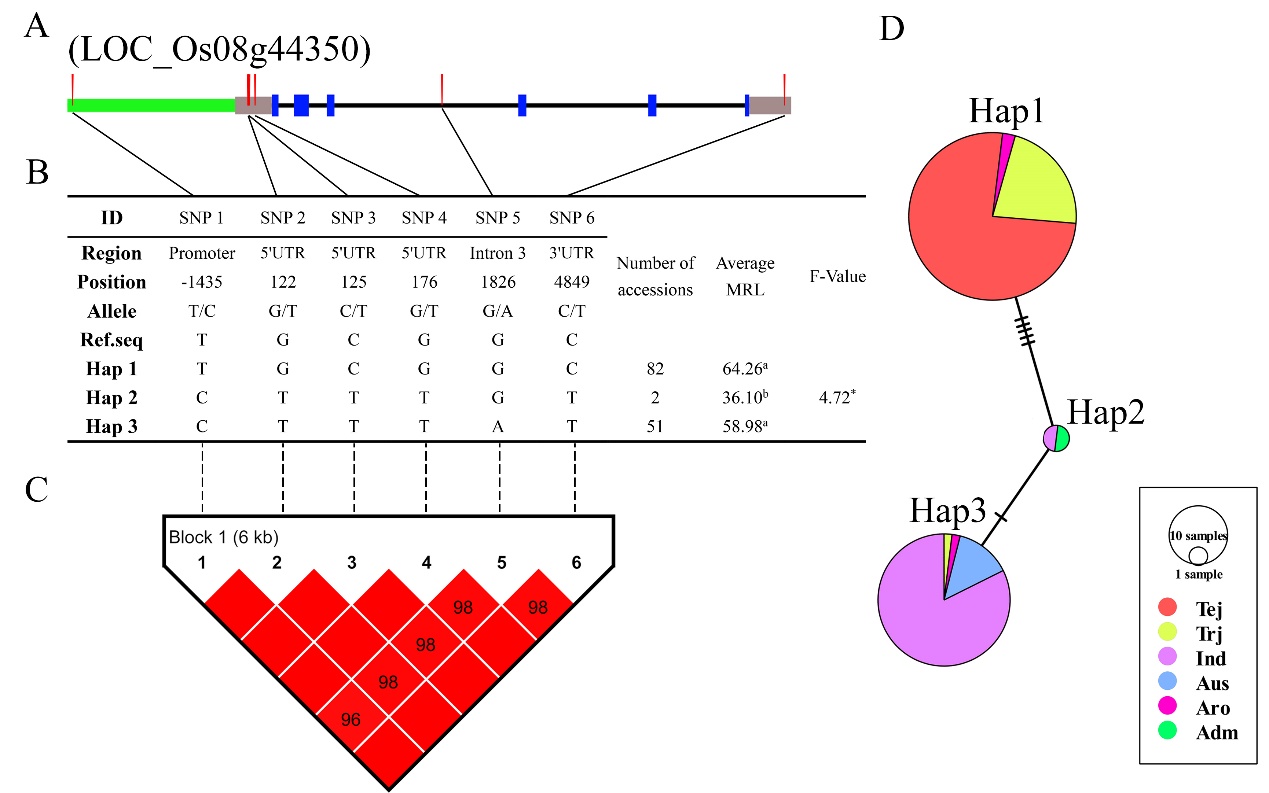


**Figure 4.** Haplotype analysis of *LOC_Os08g44350*. (A) Schematic representation of gene structure and SNP positions in *LOC_Os08g44350*. Green, gray, and blue blocks represent promoter, untranslated regions, and exon regions. Solid lines indicate intron regions. Red vertical bars represent SNPs. (B) Results of haplotype analysis. Hap: haplotype. Letters: a, b represent different significant level at *P < 0.05 (Duncan test) (C) LD analysis of SNPs in *LOC_Os08g44350. D’* was used to indicate LD level, with LD blocks defined using the Confidence Intervals function in the analysis software. Red blocks indicate complete LD between each SNP. (D) Haplotype variation analysis. Colors indicate rice subspecies as indicated in the legend. Circle size indicates the number of varieties in each Hap. Traverse lines represent the extent of variation between two Haps.


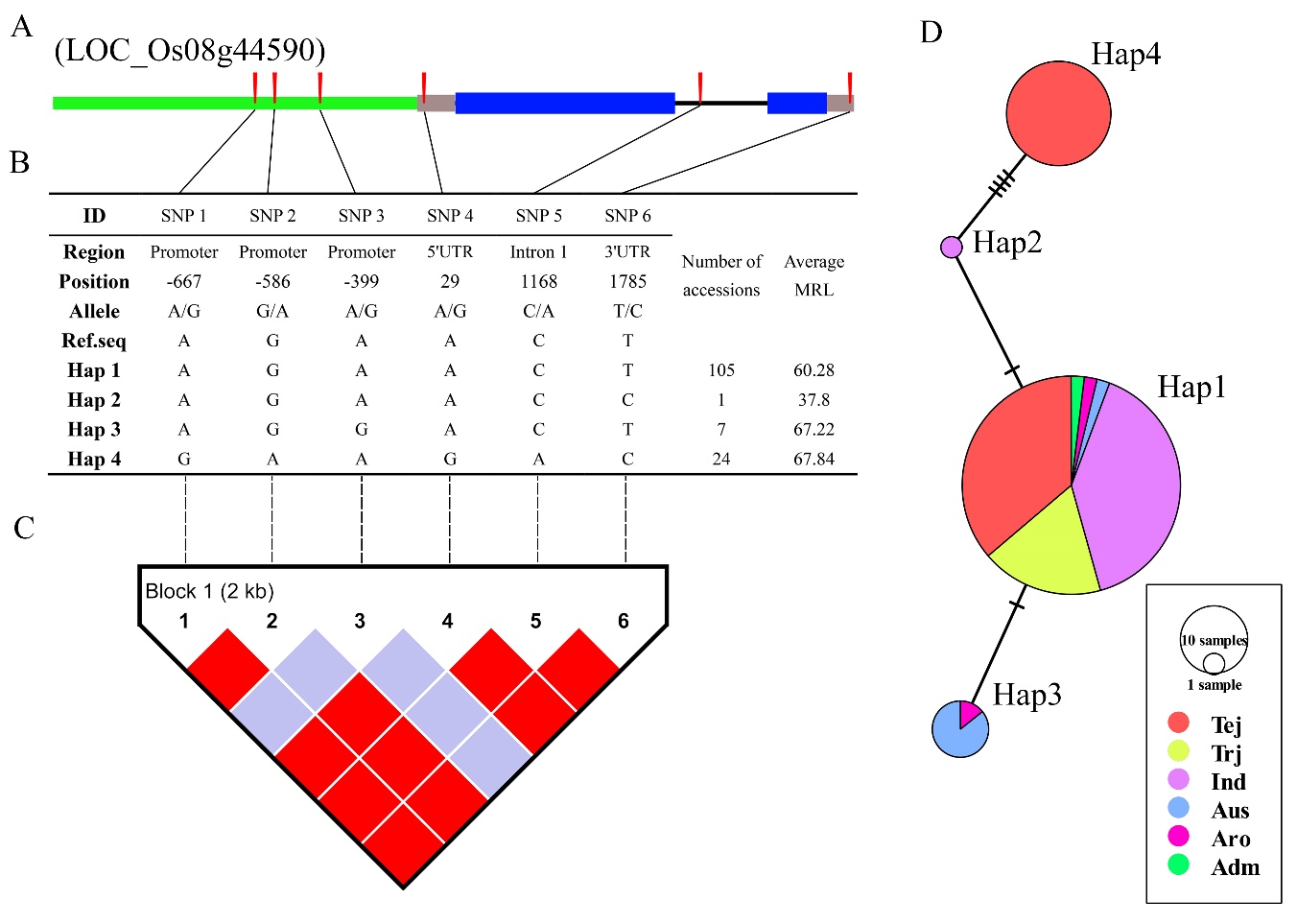


**Figure 5.** Haplotype analysis of *LOC_Os08g44590*. (A). Schematic representation of gene structure and SNP positions in *LOC_Os08g44590*. Green, gray, and blue blocks represent promoter, UTR, and exon regions. Solid lines indicate intron regions. Red vertical bars represent SNPs. (B). Results of haplotype analysis. Hap: haplotype. (C). LD analysis of SNPs in *LOC_Os08g44590*. *D*’ was used to indicate LD level, with LD blocks defined using the Confidence Intervals function in the analysis software. Red blocks indicate complete LD between each SNP. (D). Haplotype variation analysis. Colors indicate rice subspecies as indicated in the legend. Circle size indicates the number of varieties in each Hap. Traverse lines represent the extent of variation between two Haps.


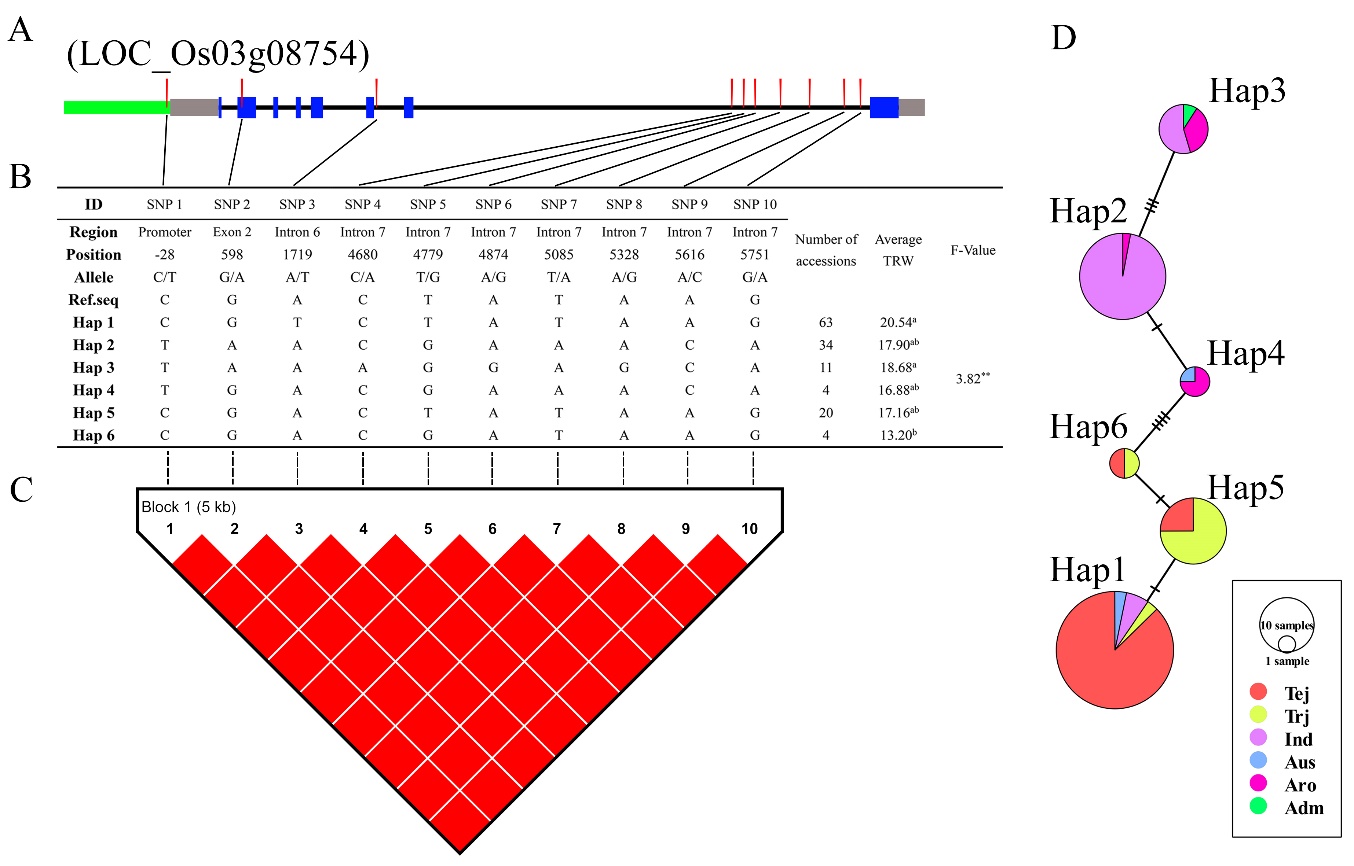


**Figure 6.** Haplotype analysis of *LOC_Os03g08754*. (A). Schematic representation of gene structure and SNP positions in *LOC_Os03g08754*. Green, gray, and blue blocks represent promoter, UTR, and exon regions. Solid lines indicate intron regions. Red vertical bars represent SNPs. (B). Results of haplotype analysis. Hap: haplotype. Letters: a, b represent different significant level at **P < 0.01 (Duncan test) (C). LD analysis of SNPs in *LOC_Os03g08754*. *D*’ was used to indicate LD level, with LD blocks defined using the Confidence Intervals function in the analysis software. Red blocks indicate complete LD between each SNP. (D). Haplotype variation analysis. Colors indicate rice subspecies as indicated in the legend. Circle size indicates the number of varieties in each Hap. Traverse lines represent the extent of variation between two Haps.


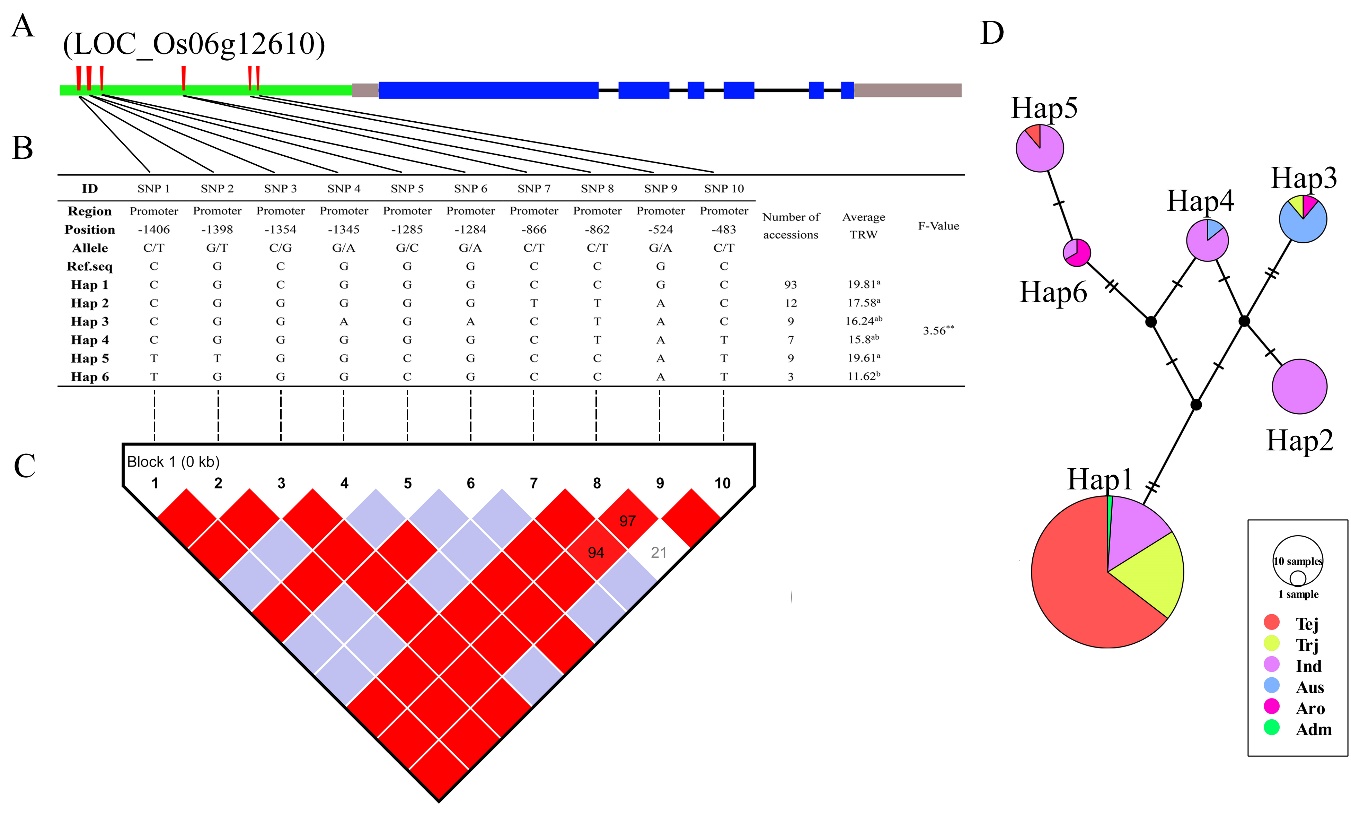


**Figure 7.** Haplotype analysis of *LOC_Os06g12610*. (A). Schematic representation of gene structure and SNP positions in *LOC_Os06g12610*. Green, gray, and blue blocks represent promoter, UTR, and exon regions. Solid lines indicate intron regions. Red vertical bars represent SNPs. (B). Results of haplotype analysis. Hap: haplotype. Letters: a, b represent different significant level at **P < 0.01 (Duncan test) (C). LD analysis of SNPs in *LOC_Os06g12610*. *D*’ was used to indicate LD level, with LD blocks defined using the Confidence Intervals function in the analysis software. Red blocks indicate complete LD between each SNP. (D). Haplotype variation analysis. Colors indicate rice subspecies as indicated in the legend. Circle size indicates the number of varieties in each Hap. Traverse lines represent the extent of variation between two Haps.
